# Facile Synthesis of Chromium-Doped Fe_1.1_Mn_1.9_O_4_ Nanoparticles and the Effect of Cr Content on Their Magnetic and Structural Properties

**DOI:** 10.3390/nano13152203

**Published:** 2023-07-29

**Authors:** Aleksandr A. Spivakov, Li-Huai Huang, Ying-Zhen Chen, Chun-Rong Lin

**Affiliations:** Department of Applied Physics, National Pingtung University, No. 4-18 Minsheng Rd., Pingtung County, Pingtung City 90003, Taiwan

**Keywords:** Fe_1.1_Mn_1.9_O_4_ nanoparticles, chromium doping, magnetic properties, combustion method, spinel ferrites, high-temperature magnetic measurements

## Abstract

In the present study, Fe_1.1_(Cr_x_Mn_1-x_)_1.9_O_4_ nanoparticles (0 ≤ x ≤ 0.5) were successfully synthesized by a combustion method, and the influence of Cr substitution on the structural and magnetic properties of the obtained nanoparticles was studied by various methods. The structural analysis revealed that the sample with x = 0 has a tetragonal structure, while all Cr-doped samples crystallize into a cubic structure. Additionally, the results of TEM show that doping with chromium leads to an increase in particle size. The magnetic hysteresis loops demonstrate the behavior typical for soft magnetic materials with low coercivity and remanence magnetization. The magnetic measurements revealed that the saturation magnetization of the obtained nanoparticles demonstrates a decreasing trend with increasing Cr content. The influence of chromium doping on the observation change in saturation magnetization is discussed. Based on the results of temperature-dependent magnetization measurements, it was found that the temperature of a magnetic transition in synthesized nanoparticles depends on Cr content.

## 1. Introduction

The Mn_x_Fe_3-x_O_4_ system has been actively studied over a long period [1,2] due to specific physical properties depending on its composition and great practical potential in such areas as biomedicine, lithium-ion batteries, water treatment, and supercapacitors [3,4,5,6,7,8]. This system can crystallize into two different structures: a cubic structure at x < 1.9 and a tetragonally deformed spinel structure at x > 1.9 (for bulk and single-crystal samples) [9,10]. The cubic–tetragonal transition is related to the cooperative Jahn–Teller effect, which is associated with the orientation of the tetragonally distorted ${Mn}^{3+}O_{6}^{2-}$ octahedra. As *x* increases, the concentration of distorted octahedra increases, and at a critical concentration, the tendency of neighboring Mn^3+^ octahedra to be elongated in the same direction leads to a transition to a tetragonal structure [11,12]. Moreover, studies have shown that the distribution of cations in the Mn_x_Fe_3-x_O_4_ system depends on its composition and can be approximately expressed by the following formulas: $\left({Mn}_{x}^{2+}{Fe}_{1-x}^{3+}\right)[{Fe}_{1-x}^{2+}{Fe}_{1+x}^{3+}]O_{4}^{2-}$ when 0 ≤ x < 1 [13] and $\left({Mn}^{2+}\right)[{Fe}_{3-x}^{3+}{Mn}_{x-1}^{3+}]O_{4}^{2-}$ or $\left({Mn}_{1-y}^{2+}{Fe}_{y}^{3+}\right)[{{Mn}_{y}^{2+}{Mn}_{x-1}^{3+}Fe}_{3-y-x}^{3+}]O_{4}^{2-}$ for x ≥ 1 [14,15], where round brackets and square brackets denote the tetrahedral and octahedral sublattices, respectively. Since the properties of spinel ferrites depend on the synthesis method, many different synthesis techniques of Mn_x_Fe_3-x_O_4_ have been described in the literature, including sol–gel, combustion, hydrothermal, and other techniques [16,17,18,19,20]. Among these methods, the combustion method has shown promising results for obtaining high-purity and uniformly sized nanoparticles [21,22]. Additionally, this method does not require sophisticated equipment and is simple and energy-efficient. 

Additional interest in studying spinel ferrites is due to the possibility of tailoring the properties by cation substitution, increasing their practical application opportunities. In particular, the influence of various cations on the properties of Mn_x_Fe_3-x_O_4_ nanoparticles has been discussed in the literature, including the effect of La and Sm on photocatalytic properties [23,24]; Mo, Cr, and Zn on dielectric properties [25,26,27,28]; Cu, Co, and Dy on magnetic properties [29,30,31,32]; and so on. However, most studies have discussed the effect of substitution on the properties of the Mn_x_Fe_3-x_O_4_ system in the iron-rich region, especially on the properties of manganese ferrite, while the literature data on the study of the manganese-rich region (x~2) are limited. In this regard, the development of synthesis methods and the study of the effect of substitution on the structural and physical properties of Mn_x_Fe_3-x_O_4_ nanoparticles in the manganese-rich region is an important task, since the results obtained can be used to further develop the practical application of such nanoparticles. Since Cr^3+^ ions prefer to occupy octahedral positions, the choice of chromium for doping makes it possible to tailor the magnetic properties for various technological applications [33]. Additionally, the effects of chromium on improving the properties of zinc lanthanum nanoferrites for high-frequency microwave absorption [34], improving the temperature stability of nickel-zinc ferrites [35], the application potential of chromium-doped CoFe_2_O_4_ nanoparticles in magnetic fluids and microwave-absorbing applications [36], etc., have been discussed in the literature. All this makes it an exciting prospect to study the effect of chromium on the structural and physical properties of Fe_1.1_Mn_1.9_O_4_ nanoparticles.

In light of this, in the present work, we focused on the synthesis of Cr-doped Fe_1.1_Mn_1.9_O_4_ nanoparticles by combustion method and the study of the effect of chromium doping on the structural and physical properties of obtained nanoparticles by various methods. The influence of high-temperature magnetic measurements on the synthesized samples was also studied.

## 2. Experimental

### 2.1. Synthesis of Cr-Doped Fe_1.1_Mn_1.9_O_4_ Nanoparticles 

In the present work, Fe_1.1_(Cr_x_Mn_1-x_)_1.9_O_4_ nanoparticles (0 ≤ x ≤ 0.5) were prepared by the combustion method. All the reagents were of analytical grade and were used without further purification. In a typical process, stoichiometric quantities of manganese (II) nitrate tetrahydrate (Mn(NO_3_)_2_·4H_2_O), chromium (III) nitrate nonahydrate (Cr(NO_3_)_3_·9H_2_O), and iron (III) nitrate nonahydrate (Fe(NO_3_)_3_·9H_2_O) were dissolved in a solution of citric acid, glycine, and deionized water. The obtained solution was then heated to 130 °C under magnetic stirring to form the gel. After the solution became homogeneous, the temperature was raised to 200 °C for 10 min. The gel ignited spontaneously and formed an aggregate of loose powders. When the reaction was completed, the obtained precursor was placed in an oven heated to 200 °C and annealed at T_A_ = 1100 °C for 1 h. After the completion of the annealing process, the samples were quenched in air. 

### 2.2. Characterizations

The structural properties of the obtained samples were characterized by X-ray powder diffraction (XRD) using a Bruker D8 Advance diffractometer (Cu Kα radiation, 40 kV, 25 mA, λ = 1.5418 Å). The morphology of the nanoparticles was characterized by transmission electron microscopy (TEM) using the JEOL JEM-1230 transmission electron microscope operated at an accelerating voltage of 120 kV. The magnetic properties of the synthesized samples were examined via a vibrating sample magnetometer Lakeshore 7400 series VSM in the applied field of H = ±18 kOe.

## 3. Results and Discussion

### 3.1. Structural Analysis

**Figure 1:**
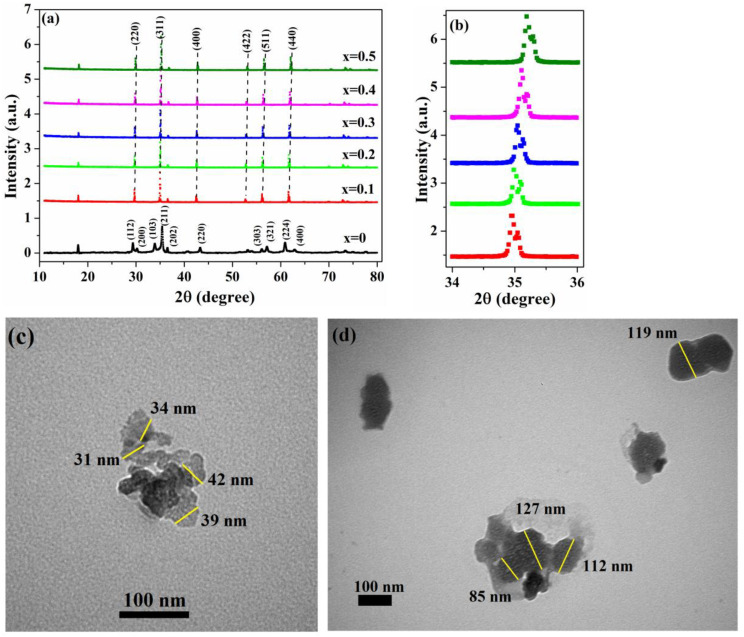
(**a**) XRD patterns of the Fe_1.1_(Cr_x_Mn_1-x_)_1.9_O_4_ nanoparticles with 0 ≤ x ≤ 0.5; (**b**) shifting of (311) peak for samples with 0.1 ≤ x ≤ 0.5; (**c**) and (**d**) TEM images of the samples with x = 0 and x = 0.5, respectively.

Figure 1a shows the X-ray diffraction (XRD) patterns of the samples with various chromium concentrations. 

The peak positions for the sample with x = 0 at 18, 29.4, 30.2, 33.9, 35.4, 36.5, 43.3, 56, 57.2, 60.9, and 62.9 correspond to a tetragonal spinel structure of FeMn_2_O_4_ [37]. However, for Cr-doped samples, the diffraction peaks match the indexed planes (220), (311), (400), (511), (440), (533), and they agree with a face-centered cubic structure of jacobsite ferrite [38] (JCPDS Card No. 10–0319) with space group $Fd\bar{3}m$. The obtained XRD patterns demonstrate that no synthesized samples contain features of any impurities. As noted in the introduction, the cubic–tetragonal transition in the Mn_x_Fe_3-x_O_4_ system is related to the increase in Mn^3+^ ions in octahedral sites above a critical concentration. Therefore, the formation of a cubic structure in our samples can be related to the fact that the substitution of manganese ions by smaller chromium ions leads to a decrease in the number of distorted ${Mn}^{3+}O_{6}^{2-}$ octahedra and, as a result, to the formation of a cubic structure in Cr-doped samples.

The Scherrer equation was used to calculate the average crystallite size (D_XRD_) of the synthesized samples, and the lattice parameters of the samples were estimated using Bragg’s law and the d-spacing equations for tetragonal and cubic structures. The obtained values are summarized in Table 1.

The calculated values of lattice parameters for the Fe_1.1_Mn_1.9_O_4_ sample are close to experimental and theoretically calculated values for FeMn_2_O_4_ [10,37,39]. For the samples with x = 0.1–0.5, the results obtained demonstrate that the value of lattice parameter *a* decreases with the increase in Cr content. Furthermore, as can be seen from Figure 1b, the increase in Cr content leads to a shift in the position of (311) peak towards higher values of 2θ. The results of previous studies of cation-substituted spinel oxides [40,41] have shown that both of these results can be explained by the substitution of larger Mn ions (r_Mn_ = 0.645 Å) by smaller Cr ions (r_Cr_ = 0.615 Å) on the octahedral sites. Moreover, the shift in the (311) peak toward higher angles implies [42] that Cr was incorporated into the crystal lattice of Fe_1.1_Mn_1.9_O_4_. 

The TEM image of the Fe_1.1_Mn_1.9_O_4_ nanoparticles is shown in Figure 1c and demonstrates that synthesized nanoparticles have a quasi-spherical shape and are almost uniform in size. The observed agglomeration of nanoparticles in the sample may be associated with the effect of Van der Waals forces that dominates all other forces when the particle size is less than a few micrometers [43]. The particle size of the sample ranges between ~25 and 45 nm with an approximate average size of 36 nm. For chromium-doped nanoparticles (see Figure 1d), an increase in both particle size and particle size distribution is observed, while the nanoparticle agglomeration level decreases. For the sample with x = 0.5, the particle size oscillates in the range of 70–135 nm with an average size of 115 nm. In addition, the particle size estimated from the TEM analysis reasonably agrees with the crystallite size calculated by the XRD analysis, which indicates the monodispersity of the synthesized samples. 

### 3.2. Magnetic Measurements

**Figure 2:**
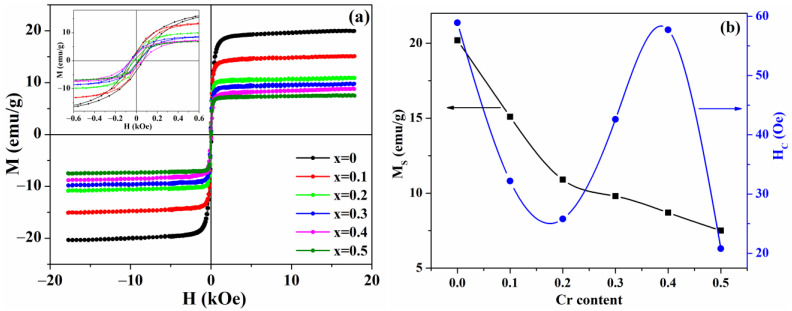
(**a**) Magnetic hysteresis loops of the Fe_1.1_(Cr_x_Mn_1-x_)_1.9_O_4_ nanoparticles; (**b**) concentration dependencies of saturation magnetization and coercivity. The inset demonstrates the hysteresis loops on an enlarged scale.

Room-temperature field-dependent magnetization curves of the samples are presented in Figure 2a, and the dependencies of saturation magnetization (M_S_) and coercivity (H_C_) on chromium concentration are shown in Figure 2b. The values of saturation magnetization, coercivity, and remanent magnetization were obtained from the M-H curves and are summarized in Table 2. The hysteresis loops of the samples demonstrate ferrimagnetic behavior with, as follows from Figure 2b and Table 2, low coercivity and remanence magnetization typical for soft magnetic materials.

The results obtained reveal that the saturation magnetization decreases with increasing Cr concentration, and this behavior of M_S_ can be described in terms of Neel’s theory. According to the theory, the net magnetization of spinel ferrites is proportional to the difference between the magnetic moment of tetrahedral (M_A_) and octahedral (M_B_) sites and can be expressed as $M_{S}=M_{B}-M_{A}$ [44]. As discussed in the introduction, in the Mn_x_Fe_3-x_O_4_ system with x ≥ 1, all Mn^3+^ ions occupy octahedral sites. Since Cr^3+^ ions strongly prefer to occupy octahedral sites [45], they will replace manganese ions in octahedral positions. Considering that the atomic magnetic moment of Cr^3+^ (3 µB) is less than the magnetic moment of Mn^3+^ (4 µB) [45,46], an increase in the chromium content will lead to a decrease in the resulting magnetization due to a weakening of the tetrahedral magnetic moment M_B_, which agrees with the experimental results.

Since the crystallite size of Cr-doped samples is higher than the limit of a single-domain region [47], a decrease in coercivity is expected with an increase in crystallite size [48], which is observed for the samples with x = 0.1 and x = 0.2. The relatively high increase in coercivity compared to the increase in crystallite size for samples with x = 0.3 and x = 0.4, as well as the sudden decrease in coercivity for a sample with x = 0.5, can be related to several factors. (1) One factor is the redistribution of cations between tetrahedral and octahedral sites with increased Cr content above 0.2 [36]. (2) Another is the effect of defects that can be formed during the synthesis of nanoparticles on the magnetocrystalline anisotropy and, as a consequence, on the coercivity [49,50]. Moreover, the influence of defects can have different effects: they can (a) decrease the coercivity if they act like centers for the formation of new domain walls, which will contribute to magnetization reversal and decrease the energy of magnetocrystalline anisotropy, or (b) increase the coercivity when the defects pin the already existing domain walls. In this case, they create local energy barriers that prevent wall movement and magnetization reversal, which increases the energy of magnetocrystalline anisotropy [51]. It is assumed that the combined effect of these factors affects the observed changes in coercivity.

### 3.3. Temperature-Dependent Magnetization Measurements

**Figure 3:**
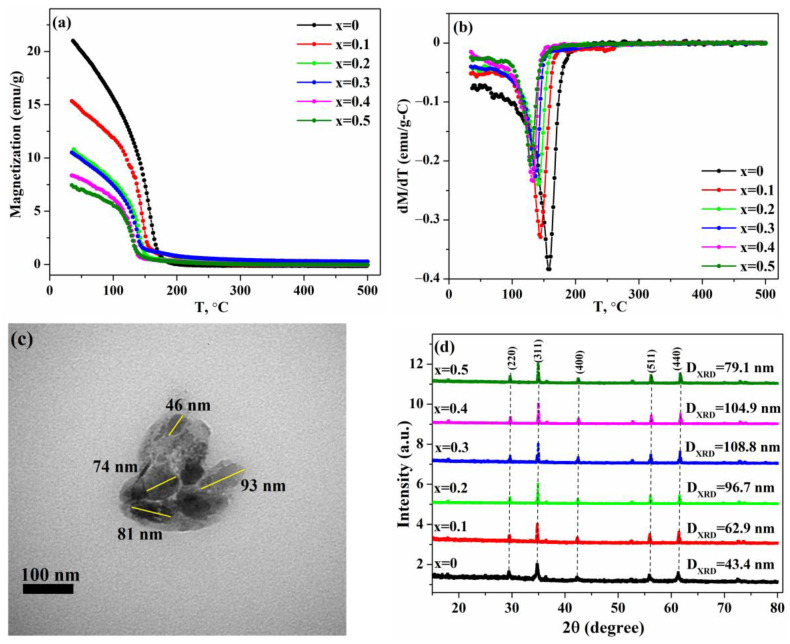
(**a**) Magnetization of the Cr–doped Fe_1.1_Mn_1.9_O_4_ nanoparticles as a function of temperature; (**b**) derivative of M with respect to temperature; (**c**) TEM image of the sample with x = 0 after high-temperature magnetic measurements; (**d**) XRD patterns of the samples after heating to 500 °C.

Temperature-dependent magnetizations of the samples heated to 500 °C are presented in Figure 3a, and dependencies dM/dT-T are shown in Figure 3b to better identify the transition temperatures. The M–T curves of all samples start from values that agree with the results of magnetic measurements of the corresponding samples, and the magnetization decreases following a Curie–Weiss type behavior. 

The analysis of the dependences dM/dT-T reveals only one anomaly at a temperature of ~140 °C, corresponding to a magnetic transition associated with ferrimagnetic–magnetic ordering [37]. However, the position of this transition in the synthesized nanoparticles is shifted towards higher temperatures compared to the single-crystal sample studied in [37]. As has been reported in studies of MnFe_2_O_4_ nanoparticles [52,53], a decrease in particle size leads to an increase in the Curie temperature compared to bulk samples due to the finite-size effect or also a non-equilibrium cation distribution, and both of these effects can affect the observed shift in the magnetic transition in the synthesized nanoparticles. It is also interesting to note that the results obtained show that the temperature of this transition depends on the concentration of chromium and shifts towards lower temperatures with increasing x. As reported previously [33], an increase in Cr content leads to a decrease in Curie temperature in MgCr_x_Fe_2−x_O_4_ samples. Since the results obtained in this work indicate that the temperature of the magnetic transition decreases with increasing chromium content, it can be assumed that the Curie temperature in Fe_1.1_(Cr_x_Mn_1-x_)_1.9_O_4_ nanoparticles also decreases with increasing Cr content. Thus, obtained nanoparticles may have the potential to be used in switches operating in a given temperature range with a tunable Curie temperature [54].

To study the effect of high-temperature measurements on the structural properties of the synthesized nanoparticles, the samples were again subjected to XRD and TEM analyses. Figure 3c shows the morphology of the sample with x = 0 after high-temperature measurements, and it can be seen that the nanoparticles mostly retain their quasi-spherical shape with a tendency to agglomerate. However, high-temperature measurements result in an increase in particle size in the sample, which oscillates between ~45 and 100 nm with an average size of about 79 nm. The XRD patterns of the samples after high-temperature magnetic measurements are shown in Figure 3d, and the results obtained demonstrate that the diffraction peaks of all samples correspond to the cubic structure of jacobsite ferrite. Thus, high-temperature measurements led to a structural transition in the Fe_1.1_Mn_1.9_O_4_ sample. Since the distribution of cations depends on the particle size [55,56,57], and the results of the TEM analysis indicate an increase in the average particle size for this sample, this transition can be associated with a redistribution of cations in the sample owing to the size effect. Moreover, the obtained XRD data also demonstrate that no samples contain features of any impurities, indicating that the synthesized samples are stable and do not degrade during the high-temperature measurements.

## 4. Conclusions

In conclusion, Cr-doped Fe_1.1_Mn_1.9_O_4_ nanoparticles were successfully synthesized by the combustion method, and the effect of Cr content on the structural and magnetic properties has been studied. The structural analysis revealed that Cr-doped samples have a cubic structure, while the Fe_1.1_Mn_1.9_O_4_ sample crystallizes into a tetragonal structure. The formation of the cubic structure can be associated with the decrease in the number of distorted ${Mn}^{3+}O_{6}^{2-}$ octahedra due to the substitution of manganese ions by smaller chromium ions. Additionally, the crystallite size calculated by the XRD analysis reasonably agrees with the particle sizes estimated from the TEM analysis, indicating the monodispersity of the synthesized samples. The magnetic measurements showed that the saturation magnetization of the samples decreased from 20.2 to 7.5 emu/g with the increase in the Cr content. The decrease in the saturation magnetization with increasing x can be explained by substituting Mn^3+^ ions on the octahedral position with Cr^3+^ ions with a less magnetic moment. Temperature-dependent magnetization measurements of the synthesized nanoparticles showed that the magnetic transition, which is associated with the ferrimagnetic–magnetic ordering, shifts towards higher temperatures compared to the bulk sample, and, in addition, the transition temperature decreases with increasing chromium concentration. The analysis of the samples after high-temperature magnetic measurements showed that in the sample with *x* = 0, an irreversible structural transition to the cubic phase occurs, and in all Cr-doped samples, the cubic phase is retained. Moreover, high-temperature magnetic measurements lead to an increase in particle size of the Fe_1.1_Mn_1.9_O_4_ sample, while a decrease in average crystallite size is observed for samples doped with chromium. 

## Figures and Tables

**Table 1:** The average crystallite sizes calculated by the Scherrer equation (D_XRD_), and lattice constants of the Fe_1.1_(Cr_x_Mn_1-x_)_1.9_O_4_ nanoparticles.

Concentration of Chromium, x	0	0.1	0.2	0.3	0.4	0.5
D_XRD_, nm	26.7	97.4	121.5	119.2	116.7	102.4
a, Å	5.93	8.51	8.5	8.48	8.46	8.43
c, Å	8.67	–	–	–	–	–

**Table 2:** Magnetic parameters of the Cr-doped Fe_1.1_Mn_1.9_O_4_ nanoparticles. Here, M_S_—saturation magnetization; M_R_—remanent magnetization; H_C_—coercivity; M_R_/M_S_—squareness ratio.

Cr concentration	0	0.1	0.2	0.3	0.4	0.5
M_S_, emu/g	20.2	15.1	10.9	9.8	8.7	7.5
M_R_, emu/g	3.4	2.3	1.7	1.9	2.2	0.9
M_R_/M_S_	0.17	0.15	0.16	0.19	0.25	0.12
H_C_, Oe	58.9	32.2	25.8	42.6	57.7	20.8

## Data Availability

The data presented in this study are available upon request by email: aleksandr.a.spivakov@gmail.com. The data are not publicly available because they are part of ongoing research.
